# Staged Management of Delayed‐Onset Upper Arm Compartment Syndrome Following Humeral Shaft Fracture: A Case Report

**DOI:** 10.1155/cro/2751438

**Published:** 2026-03-09

**Authors:** Ryo Tazawa, Hirokazu Ishihara, Sho Emura, Tetsuo Yoshihira, Shoichiro Terasawa, Ayumi Tsukada, Tomohiko Kanbe, Masashi Takaso

**Affiliations:** ^1^ Department of Orthopaedic Surgery, Machida Municipal Hospital, Tokyo, Japan, machida-city-hospital-tokyo.jp/; ^2^ Department of Orthopaedic Surgery, Kitasato University School of Medicine, Sagamihara, Kanagawa, Japan, kitasato-u.ac.jp

## Abstract

**Background:**

Acute compartment syndrome (ACS) is a limb‐threatening condition associated with high morbidity and a substantial risk of long‐term functional impairment. ACS most commonly develops shortly after fractures and typically affects the legs and forearms. Although ACS of the upper arm is rare, accounting for approximately 0.6% of cases, the condition can result in severe complications comparable to those observed in the leg and forearm. Therefore, early recognition and appropriate management are essential to prevent adverse outcomes.

**Case Presentation:**

We report the case of a 21‐year‐old male who developed ACS of the upper arm secondary to a humeral shaft fracture 4 days postinjury. The patient was referred to our hospital on postinjury Day 3 with mild upper arm pain but no neurological deficits. On postinjury Day 4, however, he experienced rapid worsening of swelling and severe pain. ACS was diagnosed based on elevated intracompartmental pressure. Staged management, consisting of emergent fasciotomy and external fixation with negative‐pressure wound therapy, followed by definitive fixation, successfully preserved upper arm function.

**Conclusions:**

ACS should be considered in all patients after fractures, including those involving uncommon anatomical sites, such as the humerus. Prompt diagnosis and timely surgical intervention are critical to prevent severe complications and preserve muscle function.

## 1. Introduction

Acute compartment syndrome (ACS) is a serious condition associated with high morbidity and potential for severe limb ischemia, which may necessitate amputation and lead to complications such as contracture and permanent nerve injury, including persistent motor and/or sensory deficits [1, 2]. Previous studies have demonstrated that staged management of tibial fractures complicated by ACS, consisting of immediate fasciotomy and external fixation with negative‐pressure wound therapy (NPWT), followed by definitive fixation, can result in favorable clinical results [3]. Therefore, early recognition and prompt intervention are critical for optimizing patient outcomes.

ACS most commonly occurs following fractures or crush injuries of the extremities, with the legs and forearms being the most frequently affected sites [4]. Although ACS can develop in any compartment of the upper and lower extremities, upper arm involvement is rare [5]. In addition, ACS typically presents shortly after high‐energy trauma, particularly in long bone fractures [4–6].

Here, we report a case of a 21‐year‐old male who developed ACS of the upper arm 4 days after sustaining a humeral shaft fracture. Staged management consisting of emergency fasciotomy and external fixation with NPWT, followed by definitive fixation, successfully treated the ACS and preserved muscle function.

The patient was informed of the intention to publish this case, and informed consent was obtained.

## 2. Case Presentation

A 21‐year‐old male with no prior medical history awoke with pain in his right upper arm. The patient presented to a local orthopedic clinic, where radiographs demonstrated a right humeral midshaft fracture. The mechanism of the injury was unclear because he had consumed alcohol the previous evening. Three days after the injury, he was referred to our hospital for surgical management. A long‐arm posterior plaster splint extending from the posterior shoulder to the wrist together with a sling was applied for immobilization. On the initial examination, the patient reported mild upper arm pain. The upper arm compartments were soft, and the neurovascular status was intact. Radiographs confirmed a right humeral midshaft fracture (AO/OTA 12‐A2; Figure 1). No clinical findings suggestive of ACS were present, and surgery was scheduled 7 days after the injury.

Figure 1Radiograph of the right humerus demonstrating a midshaft fracture. (a) Anteroposterior view. (b) Lateral view.

**Figure:**
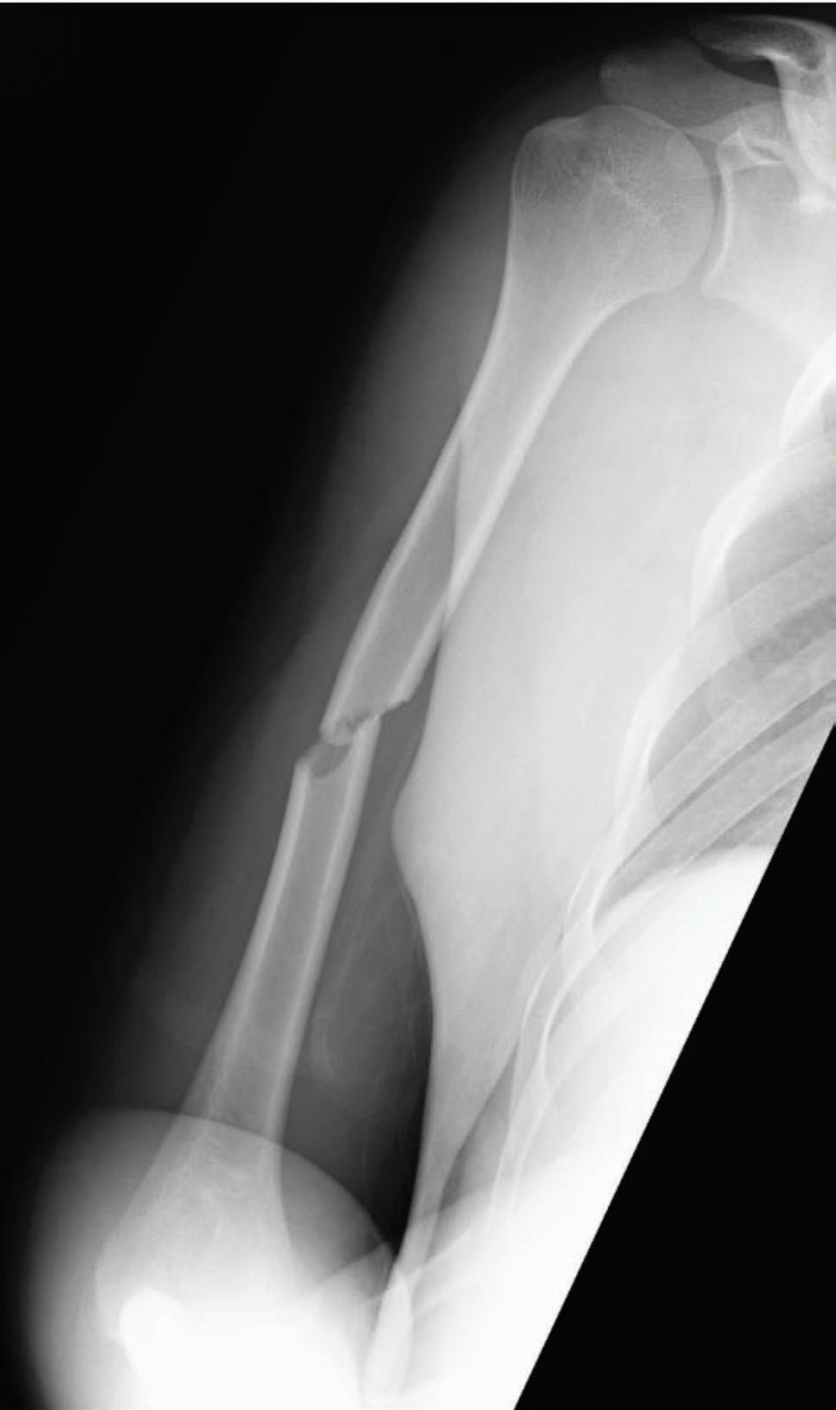
(a)

**Figure:**
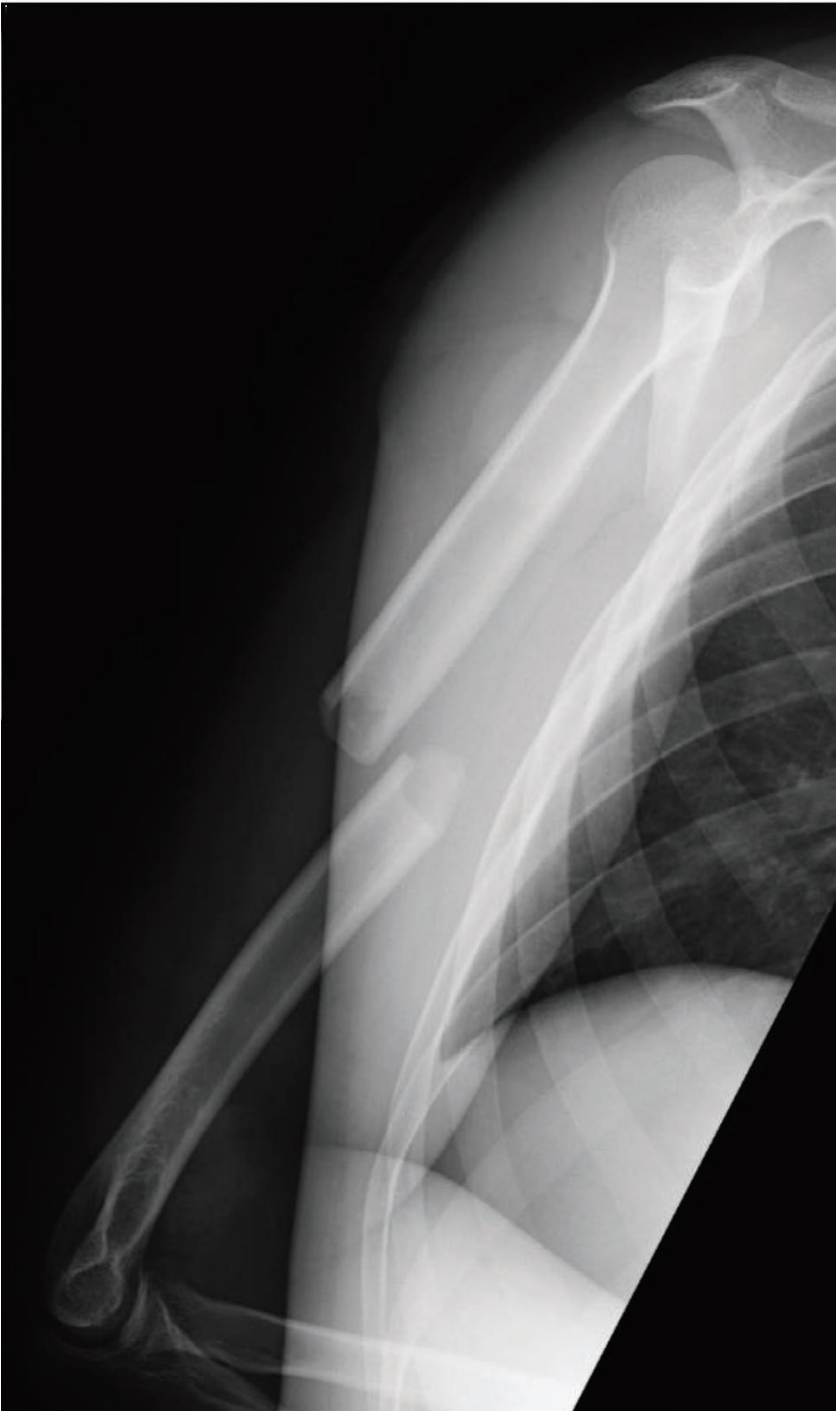
(b)

One day later, the patient returned to the emergency department with severe worsening pain in the right upper arm. Vital signs were as follows: blood pressure, 145/103 mmHg; heart rate, 75 beats/min; respiratory rate, 24 breaths/min; and body temperature, 36.1°C. The right upper arm was swollen, with edema extending to the antecubital region but not to the mid‐to‐distal forearm (Figure 2a). The patient reported severe upper arm pain, with a numerical rating scale score of 10. Radial artery pulses were palpable, and no sensory deficits were observed. Repeat radiographs showed no significant interval changes compared with those obtained at the first visit (Figure 2b,c). Immediately before fasciotomy, intracompartmental pressures were measured at the sites of maximal swelling using an 18‐gauge needle connected to an arterial pressure monitoring system and were 90 mmHg in the anterior compartment and 30 mmHg in the posterior compartment, confirming a diagnosis of ACS [7].

Figure 2(a) Clinical photograph obtained prior to fasciotomy, showing pronounced swelling of the upper arm extending to the antecubital region, with no swelling in the mid‐to‐distal forearm. Radiographs at readmission were similar to those obtained at initial presentation. (b) Anteroposterior view. (c) Lateral view.

**Figure:**
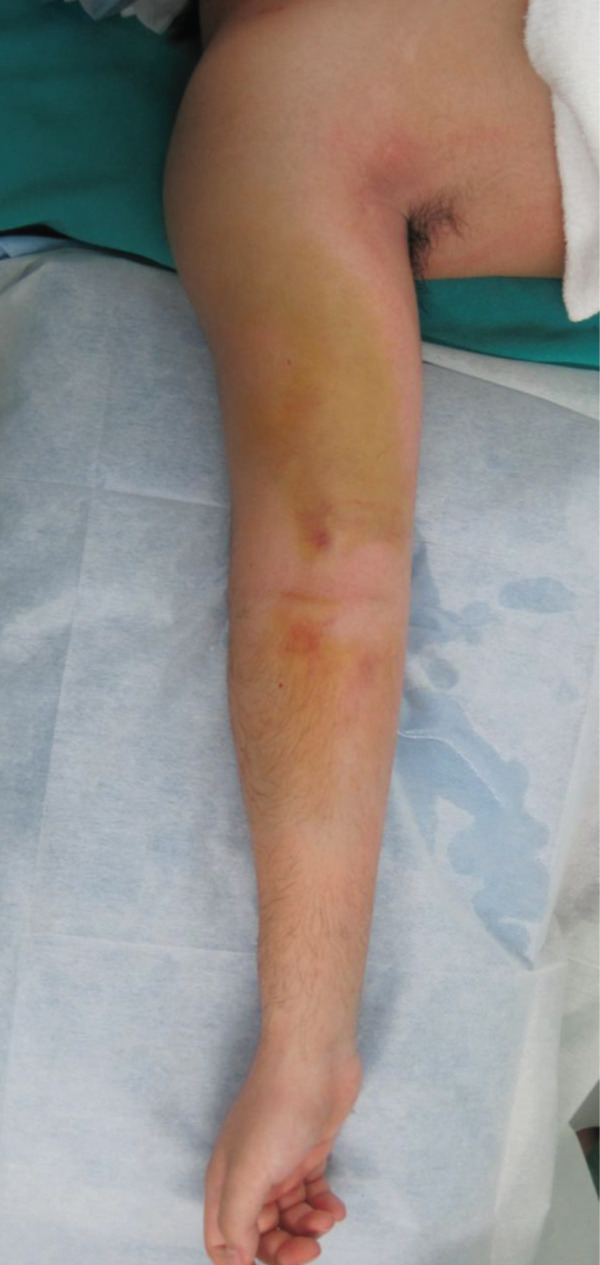
(a)

**Figure:**
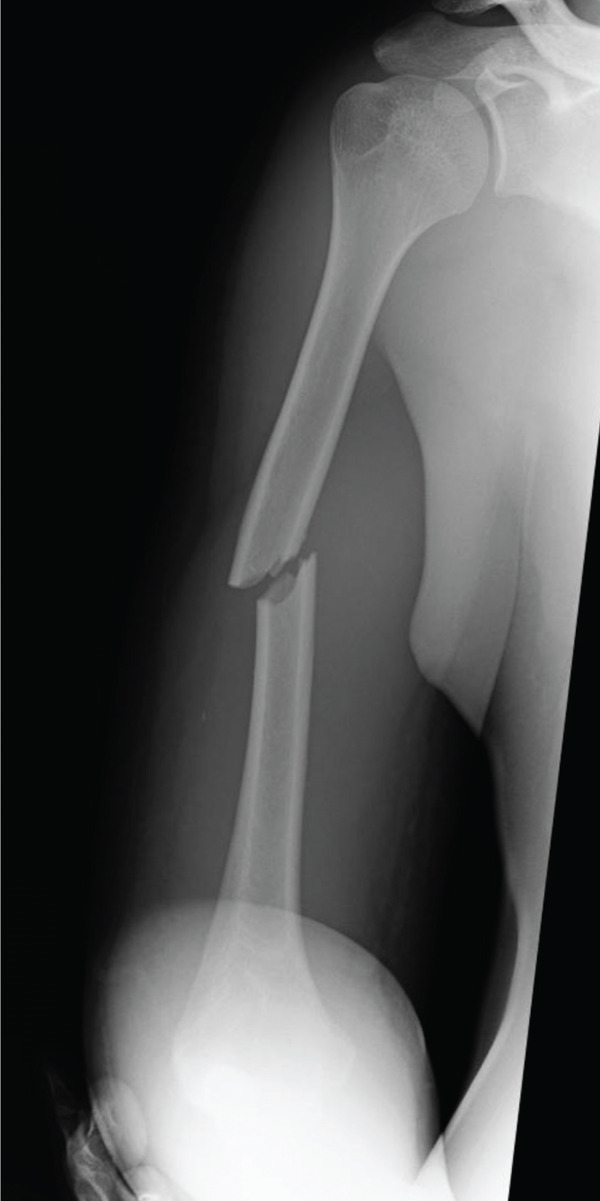
(b)

**Figure:**
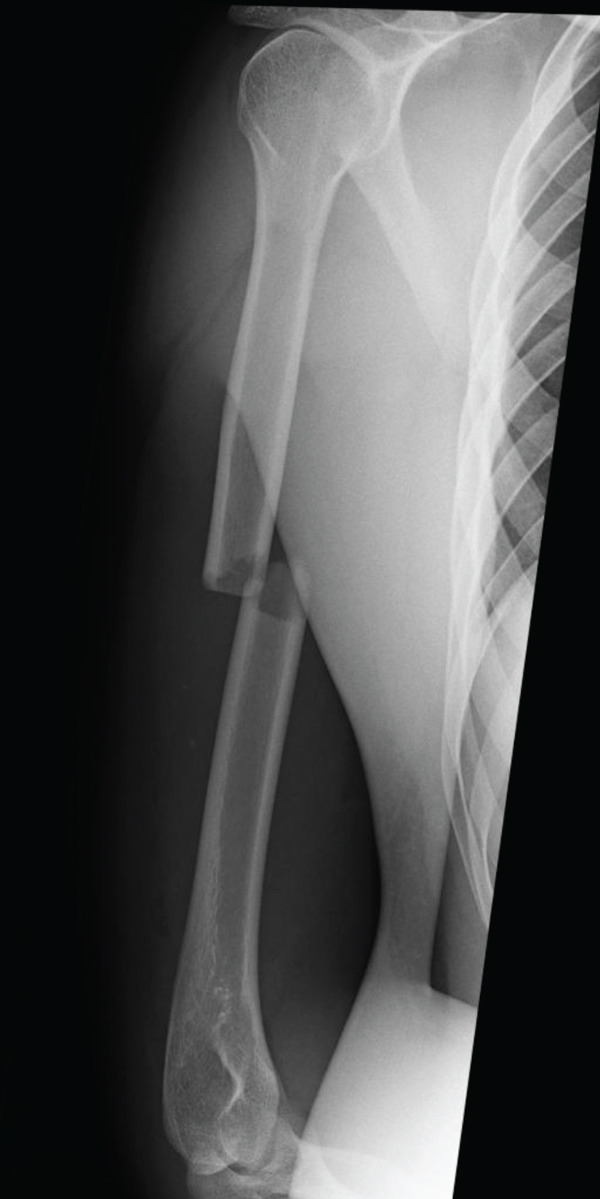
(c)

**Figure 3:**
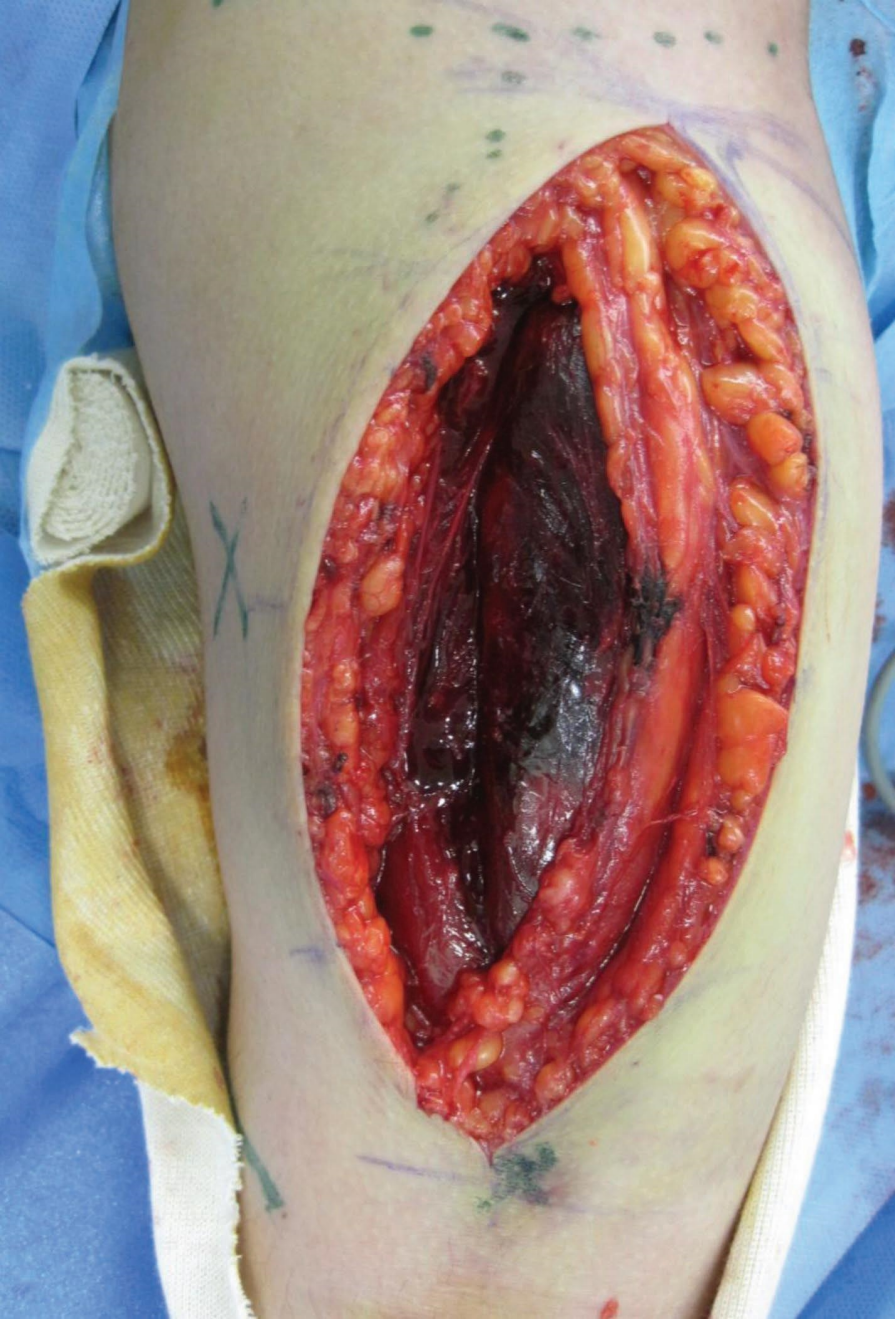
Intraoperative view following anterior fasciotomy of the right upper arm. The biceps muscle belly appeared dark red and visibly bulged from the incision site.

An emergency fasciotomy with temporary external fixation of the humeral shaft fracture was performed. A 20‐cm longitudinal skin incision was made proximally from the deltopectoral interval to the lateral edge of the biceps distally. An incision was subsequently made in the deltopectoral and biceps fascia, revealing a visibly bulging biceps muscle belly with a dark red coloration, consistent with ischemic changes (Figure 3). No vascular damage was observed. After anterior fasciotomy, repeated intracompartmental pressure measurements decreased to 18 mmHg in the anterior compartment and 8 mmHg in the posterior compartment. A temporary external fixator (DePuy Synthes, Tokyo, Japan) and a RENASYS NPWT device (Smith & Nephew, Tokyo, Japan) were applied (Figures 4a, 4b, and 4c), with a continuous negative pressure set at 40 mmHg for local damage control. At this pressure level, sufficient wound bed stimulation can be maintained while minimizing additional soft tissue stress, as an excessive pressure reduction below approximately 40 mmHg has been reported to compromise periwound mechanical stimulation [8]. The dressing was changed, and the wound was reassessed every few days. Three days postoperatively, the marked upper arm edema persisted; however, muscle discoloration substantially improved. By postoperative Day 10, both the muscle color and soft tissue edema had markedly improved, and no signs of infection were observed (Figure 5a,b). On postoperative Day 12, the swelling further decreased and the appearance of the biceps muscle normalized without evidence of infection. With recovery of muscle viability and improvement in soft tissue conditions, definitive internal fixation was performed through the same anterior approach using a 4.5/5.0‐mm narrow locking compression plate (DePuy Synthes) (Figure 6a,b). Primary skin closure was achieved without excessive tension, and a long‐arm splint was applied in the neutral position. The patient′s postoperative course was unremarkable. The splint was removed 1 week later, and light‐active and passive range‐of‐motion exercises of the shoulder and elbow were initiated. At the final follow‐up (10 months postoperatively), radiographs demonstrated healing of the humeral shaft fracture (Figure 6c,d). The patient reported no pain and had full functional recovery, with a complete range of motion (elbow flexion, 0°–135°; forearm pronation and supination, 90° each; and wrist flexion and extension, 85° each) and a Quick Disabilities of the Arm, Shoulder, and Hand score [9] of 0 points at the final follow‐up, indicating excellent upper extremity function. A summary of the patient′s diagnostic, surgical, and therapeutic management is presented in Table 1.

Figure 4(a) Application of a temporary external fixator and a negative pressure wound therapy device. Radiographs of the humerus after temporary external fixation. (b) Anteroposterior view. (c) Lateral view.

**Figure:**
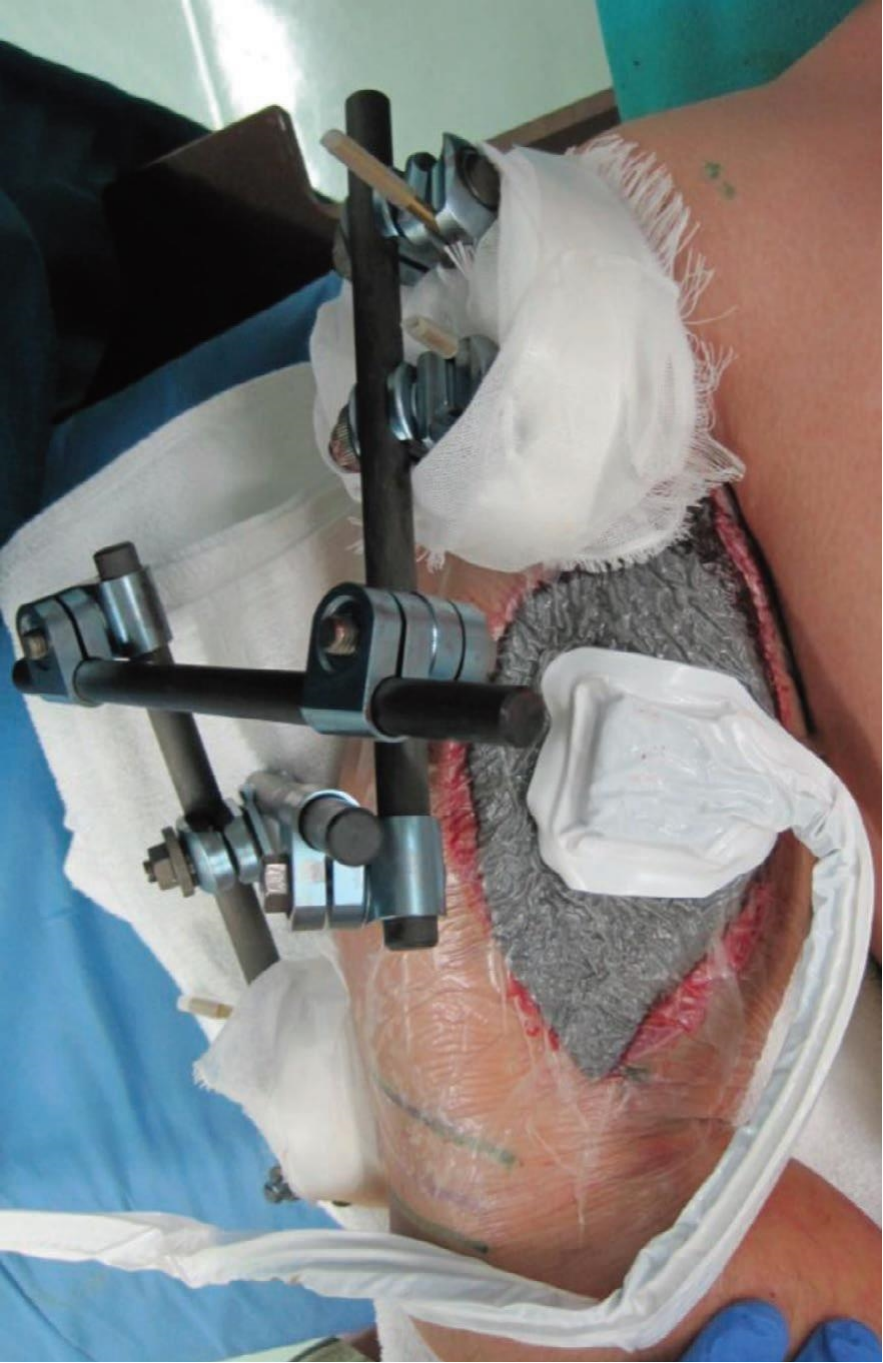
(a)

**Figure:**
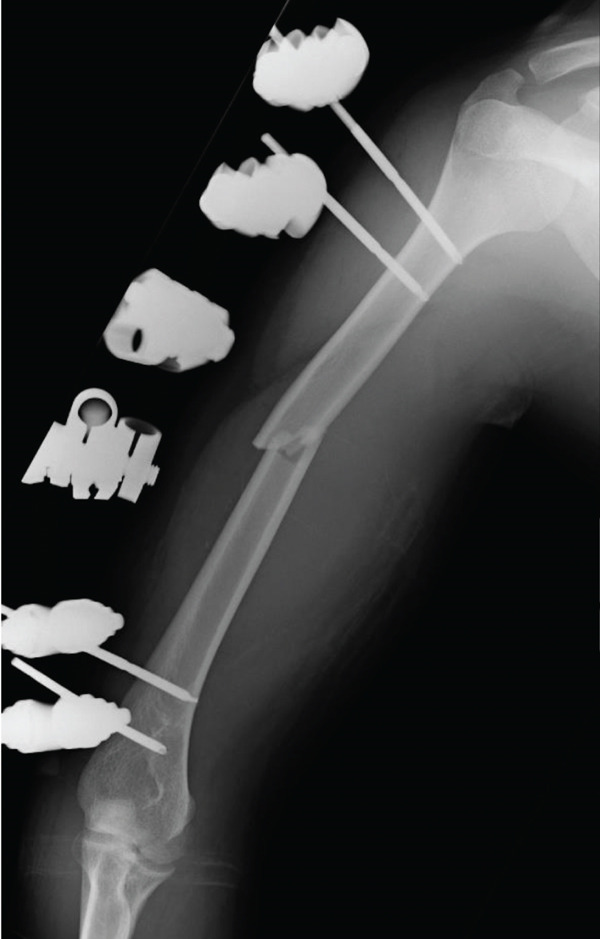
(b)

**Figure:**
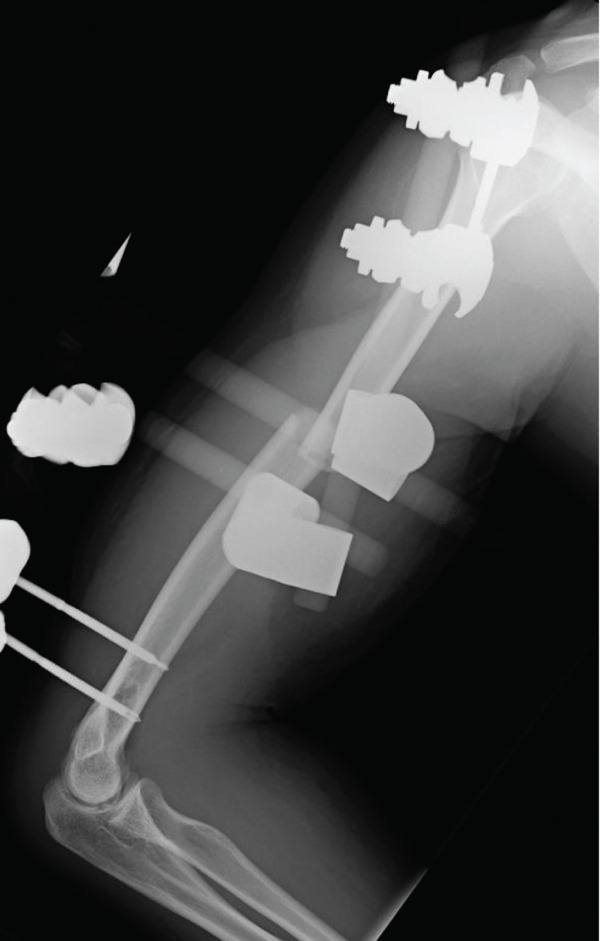
(c)

Figure 5Clinical photographs of the upper arm following fasciotomy and negative pressure wound therapy. (a) Persistent edema of the upper arm with substantial improvement in muscle discoloration 3 days postoperatively. (b) Marked improvement in both muscle color and soft tissue edema, with no signs of infection 10 days postoperatively.

**Figure:**
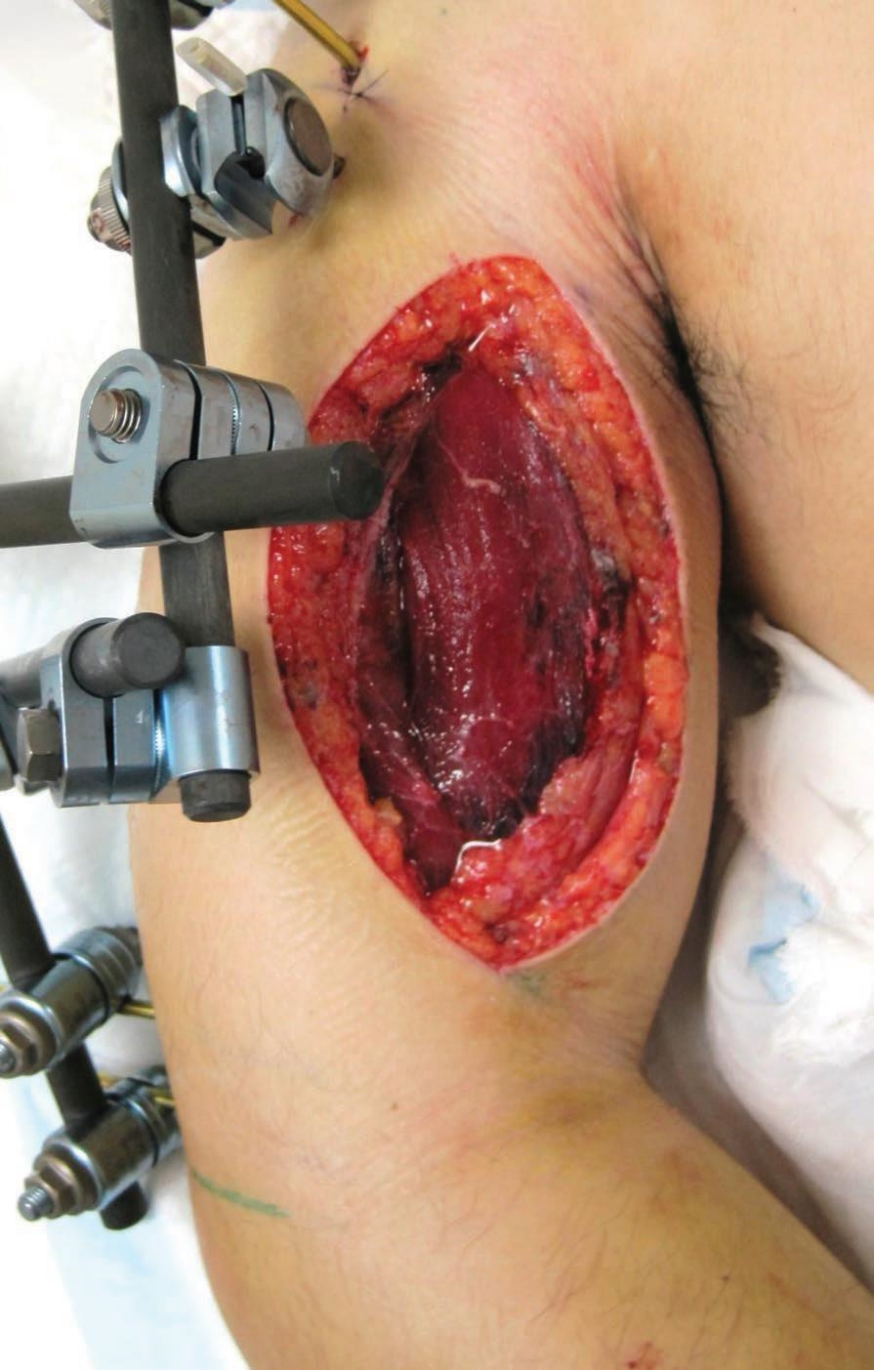
(a)

**Figure:**
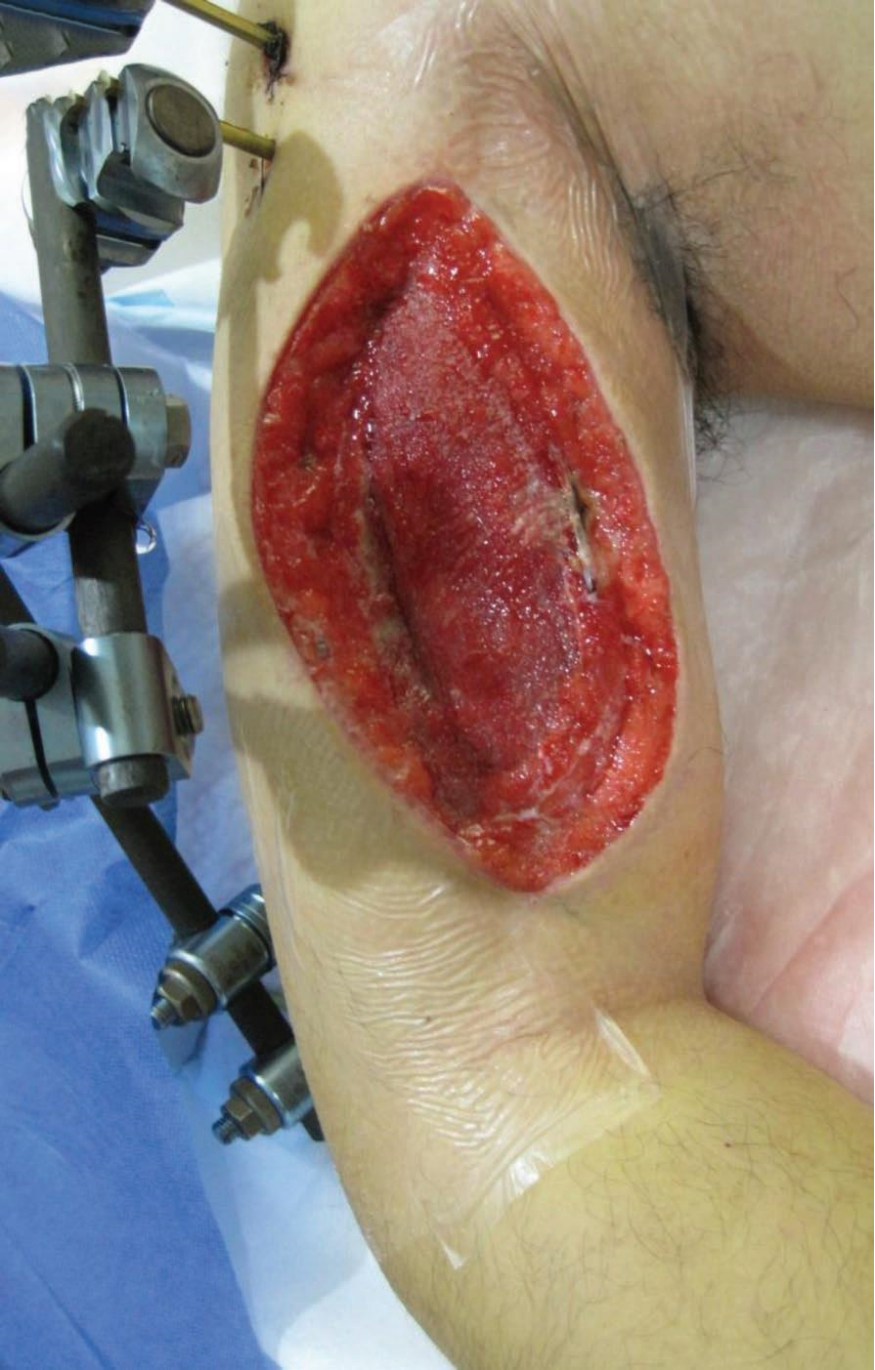
(b)

Figure 6Radiographs after definitive plate fixation of the humeral shaft fracture. (a) Anteroposterior view. (b) Lateral view. The fracture had healed 10 months postfixation. (c) Anteroposterior view. (d) Lateral view.

**Figure:**
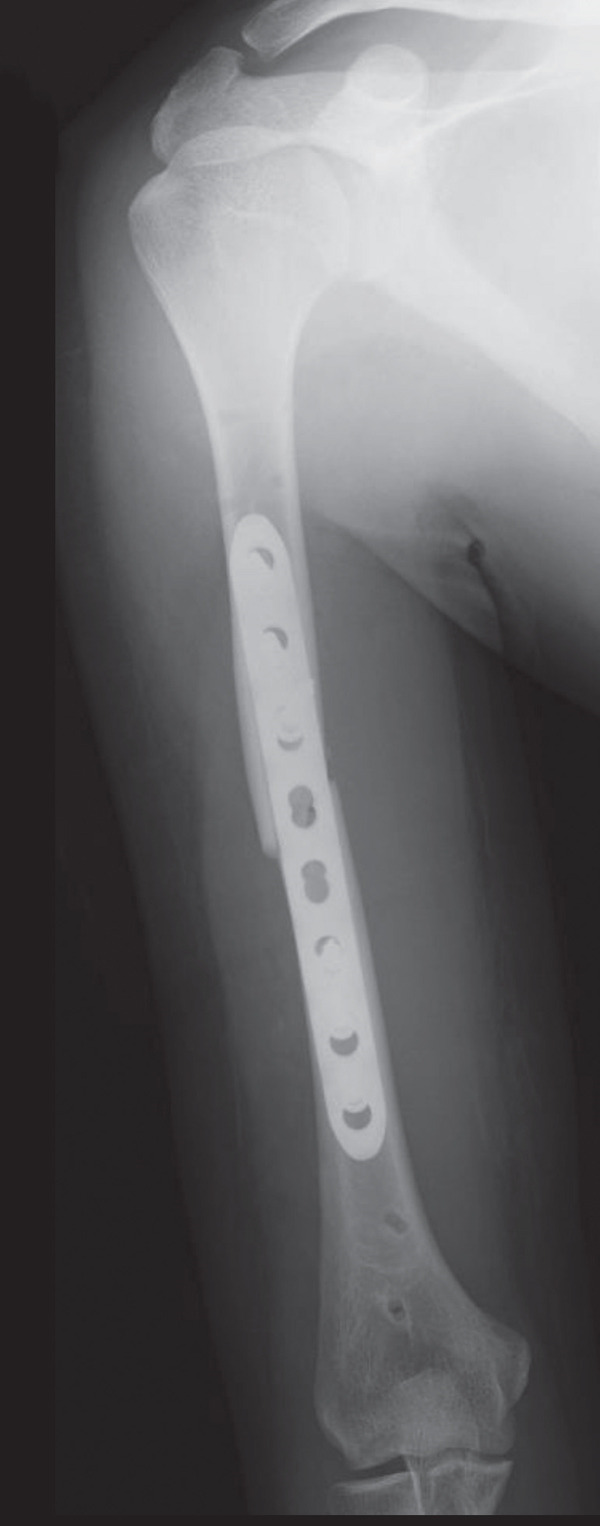
(a)

**Figure:**
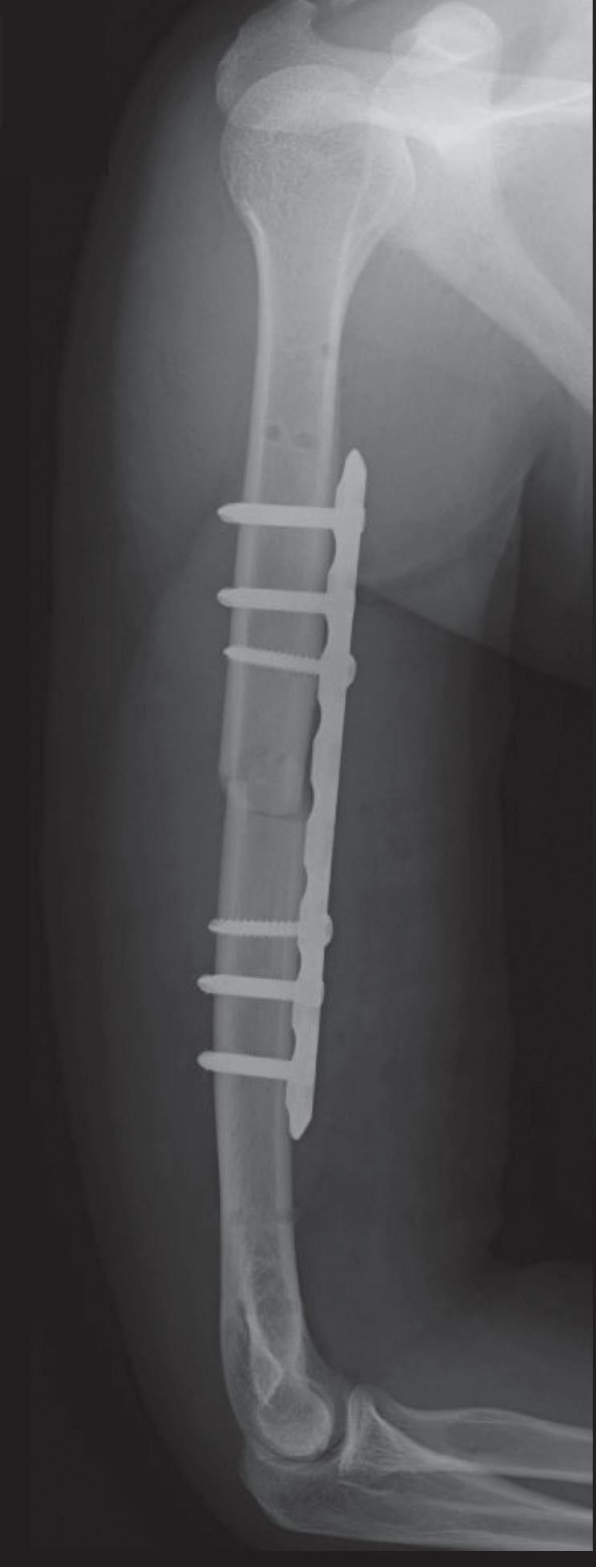
(b)

**Figure:**
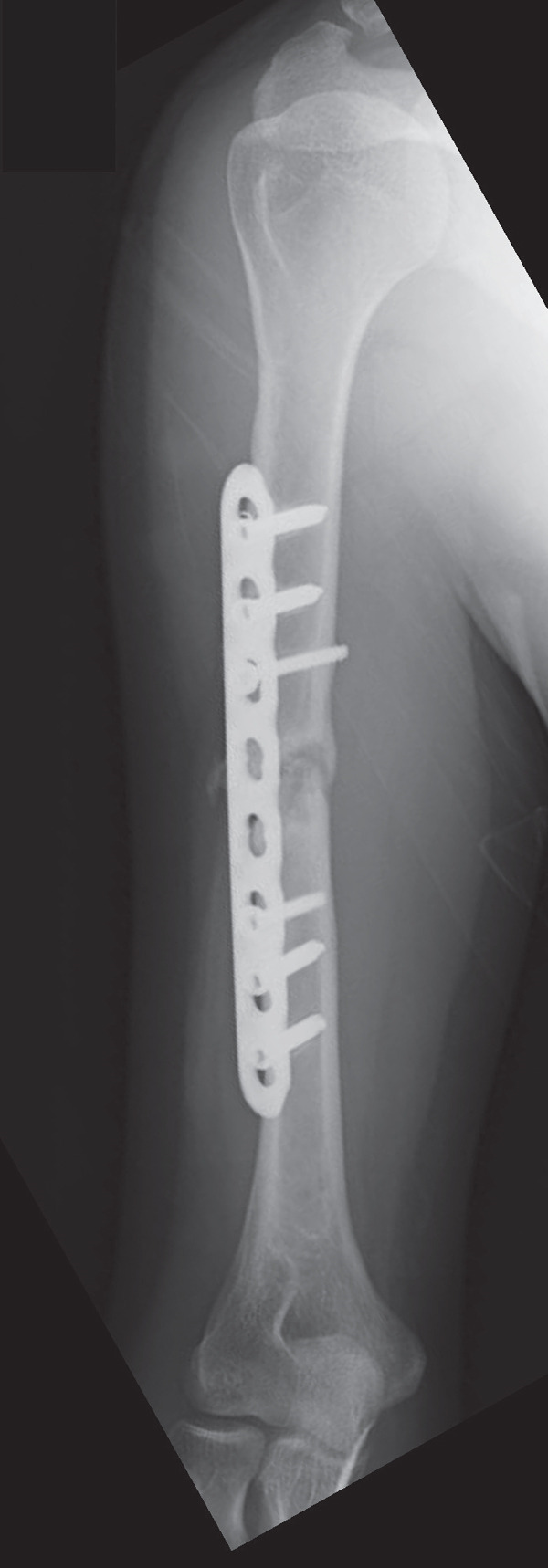
(c)

**Figure:**
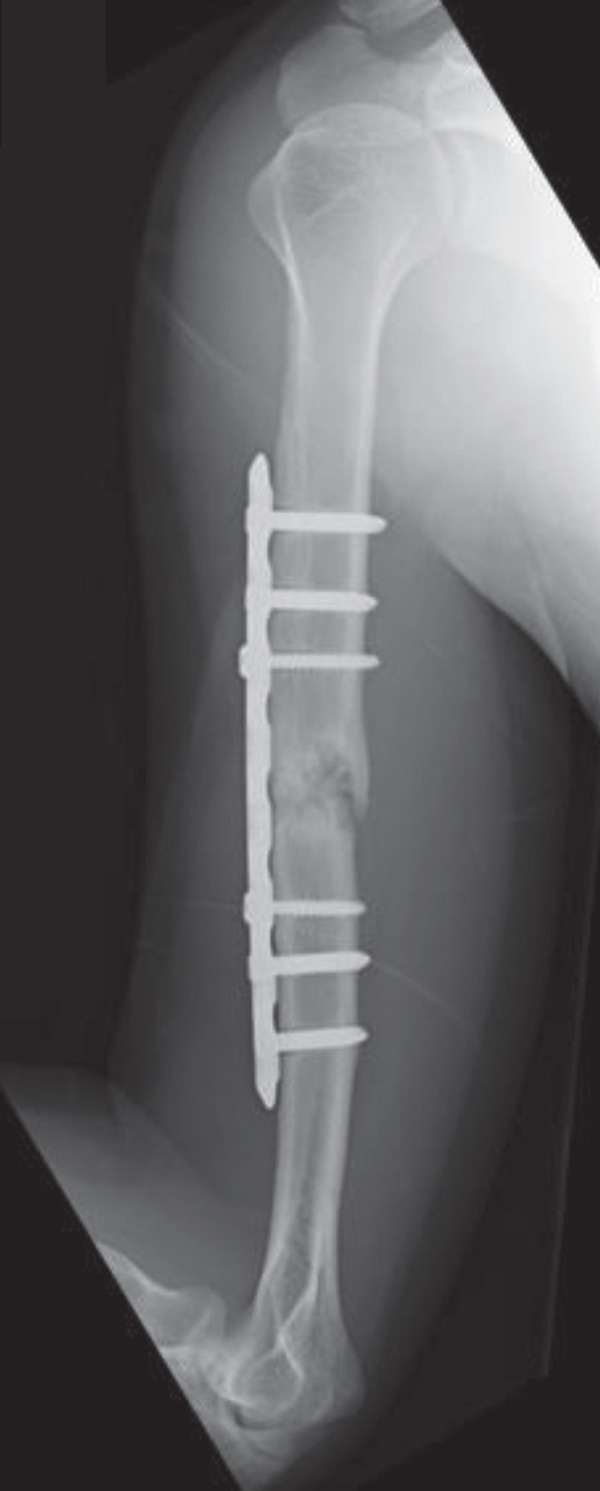
(d)

**Table 1:** Timeline of clinical events.

Time point	Clinical events
Postinjury Day 0	Injury resulting in a humeral shaft fracture; conservative management with splint immobilization in a local orthopedic clinic
Postinjury Day 3	Referred to our hospital for surgical management; mild upper arm pain and no neurological deficits
Postinjury Day 4	Progressive upper arm pain and swelling; diagnosis of acute compartment syndrome; emergency fasciotomy, temporary external fixation, and initiation of negative‐pressure wound therapy
Postoperative Day 3	Persistent edema; muscle discoloration markedly improved
Postoperative Day 10	Significant reduction of edema; no signs of infection
Postoperative Day 12	Definitive internal fixation with a locking compression plate and complete skin closure
Final follow‐up (10 months postoperatively)	Radiographic bone union confirmed; no pain, full range of motion, and complete functional recovery

## 3. Discussion

We treated a patient who developed ACS of the upper arm 4 days after sustaining a humeral shaft fracture. Prompt diagnosis, followed by staged management, including immediate fasciotomy, NPWT, and temporary external fixation, resulted in successful limb salvage and full functional recovery.

ACS most frequently occurs in the tibial diaphysis (36%), distal radius (9.8%), and forearm diaphysis (7.9%), whereas ACS following humeral fractures is rare, accounting for only 0.6% of the cases [4]. Compared with the lower leg and forearm, the upper arm and thigh contain fewer rigid ligaments and tendons and have thinner fascial compartments [10]. This anatomical difference allows the upper arm to accommodate greater swelling before intracompartmental pressure increases significantly [11], which most likely explains the delayed onset of significant symptoms in our patient.

Our case demonstrates that prompt diagnosis and immediate fasciotomy combined with NPWT and staged fracture repair can effectively preserve neurological and muscular functions while facilitating fracture union. Previous studies have reported that staged management—immediate fasciotomy and external fixation with NPWT followed by definitive fixation—reduces complications such as infection and nonunion in ACS after tibial plateau fractures [3]. NPWT has been shown to enhance wound healing by decreasing edema, promoting angiogenesis, increasing granulation tissue formation [12, 13], and preventing acute infection and osteomyelitis in open tibial fractures [14]. In this patient, NPWT facilitated skin closure during definitive fixation without infection.

However, studies on the staged management of lower extremity ACS have reported that, despite the use of NPWT, over 30% of patients ultimately require skin grafting with a mean wound closure time of approximately 9 days, indicating that NPWT primarily serves as a soft tissue conditioning method rather than a definitive wound closure strategy [15]. Similarly, a previous report of upper arm ACS treated with NPWT described progression to definitive internal fixation on hospital Day 5 but required additional soft tissue procedures, including dermal substitutes and split‐thickness skin grafting [16]. In contrast, our patient achieved spontaneous wound closure without skin grafting. This favorable outcome may be attributable to several anatomical and therapeutic factors. First, compared with the lower extremities, the upper arm possesses a thicker soft tissue envelope and a richer vascular supply, providing greater intrinsic potential for wound healing. Second, many previous studies involved high‐energy injuries with severe soft tissue compromise, which are inherently associated with poorer healing outcomes. Third, temporary external fixation in our case may have contributed to the favorable soft tissue outcome by stabilizing the fracture, minimizing micromotion, and preventing recurrent hematoma formation, thereby optimizing the local environment for NPWT‐mediated soft tissue conditioning. Importantly, staged management with NPWT and temporary external fixation in our patient achieved sufficient soft tissue stabilization to allow definitive fixation at an interval consistent with established lower extremity protocols and enabled tension‐free primary closure without requiring skin grafting. These findings suggest that appropriately timed definitive fixation combined with NPWT‐based soft tissue conditioning may reduce the need for secondary soft tissue reconstruction in upper arm ACS.

In conclusion, although ACS typically occurs shortly after significant trauma, involvement of the upper arm is rare. We report a case of ACS developing 4 days after a humeral shaft fracture that was successfully treated with staged management comprising immediate fasciotomy, external fixation with NPWT, and subsequent definitive fixation. Surgeons should maintain a high index of suspicion for ACS following fractures, even in the humerus. Prompt diagnosis and treatment are crucial for preserving muscular and neurological function.

## Funding

No funding was received for this research.

## Ethics Statement

The patient was informed that the data from this study would be submitted for publication, and informed consent was obtained.

## Conflicts of Interest

The authors declare no conflicts of interest.

## Data Availability Statement

Data sharing is not applicable to this article as no new data were generated or analyzed in this study.

## Supporting information


**Supporting Information** Additional supporting information can be found online in the Supporting Information section. File S1: CARE checklist for the reporting of this case report.
